# Efficacy and Safety of Modified Duhuo Jisheng Decoction in the Treatment of Lumbar Disc Herniation: A Systematic Review and Meta-Analysis

**DOI:** 10.1155/2020/2381462

**Published:** 2020-07-04

**Authors:** Zhencheng Xiong, Ping Yi, Liubo Zhang, Haoning Ma, Wenhao Li, Mingsheng Tan

**Affiliations:** ^1^Graduate School of Peking Union Medical College, Chinese Academy of Medical Sciences, Beijing, China; ^2^Department of Spine Surgery, China-Japan Friendship Hospital, Beijing, China; ^3^Beijing University of Chinese Medicine, Beijing, China

## Abstract

**Objective:**

Lumbar disc herniation (LDH) is based on the degenerative changes of the intervertebral disc. Many drugs are used to treat and prevent LDH, including Western medicine and Chinese medicine. Duhuo Jisheng Decoction (DHJSD) is one of the most classic Chinese medicine prescriptions. The purpose of our meta-analysis is to evaluate the efficacy and safety of modified DHJSD in the treatment of LDH.

**Methods:**

We searched multiple databases including PubMed, Embase, Cochrane Library, China National Knowledge Infrastructure (CNKI) databases, Wanfang Database, and Chinese Scientific Journal Database (VIP) to identify studies that met the inclusion criteria. This meta-analysis was registered at INPLASY with reference number ID: INPLASY202060053.

**Results:**

Fourteen randomized controlled trials (RCTs) were identified, including 1560 patients. This meta-analysis showed that the total effective rate and cure rate of modified DHJSD are higher than those of diclofenac sodium enteric-coated tablets (total effective rate: RR = 1.18, 95% CI: 1.12 to 1.25, *P* < 0.0001, *I*^2^ = 0%; cure rate: RR = 1.60, 95% CI: 1.30 to 1.97, *P* < 0.00001, *I*^2^ = 2%), diclofenac sodium enteric-coated tablets plus ibuprofen and indomethacin (total effective rate: RR = 1.23, 95% CI: 1.11 to 1.37, *P*=0.0001, *I*^2^ = 0%; cure rate: RR = 1.58, 95% CI: 1.22 to 2.04, *P*=0.0005, *I*^2^ = 0%), and diclofenac sodium sustained-release capsule (total effective rate: RR = 1.49, 95% CI: 1.27 to 1.74, *P* < 0.00001, *I*^2^ = 0%; cure rate: RR = 10.07, 95% CI: 3.29 to 30.88, *P* < 0.00001, *I*^2^ = 5%). Modified DHJSD was also better than Western medicine (MD = −1.56, 95% CI: −2.42 to −0.70, *P*=0.0004, *I*^2^ = 74%) in terms of visual analogue scale (VAS) scores. Three RCTs showed no adverse events in the modified DHJSD group, but adverse events existed in the Western medicine group.

**Conclusion:**

This meta-analysis showed that modified DHJSD had a more favorable effect on the treatment of LDH than Western medicine, and there were no obvious adverse events. More high-quality RCTs are needed to complement existing conclusions.

## 1. Introduction

Lumbar disc herniation (LDH) can lead to physical deterioration, long-term back and leg pain, neurological deficits, and socioeconomic problems due to the large medical costs associated with treatment [1]. LDH belongs to the category of “low back pain” and “Bizheng” (pain caused by wind, cold, and dampness) in traditional Chinese medicine (TCM) theory [2]. The main treatment principle of TCM is to promote blood circulation, remove blood stasis, and strengthen bones. Bizheng refers to symptoms such as joint and muscle soreness, numbness, and poor flexion and extension caused by body surface and meridian due to feelings of wind, cold, dampness, and heat [2]. Most LDH cases can achieve good results through conservative treatment, including medication, physical therapy, and steroid injection [1]. Medical treatment includes Western medicine and TCM. Western medicine mostly uses antiphlogistic and analgesic drugs for symptomatic treatment, but due to the obvious side effects of Western medicine treatment, conservative treatment of TCM was proposed [3–16]. Chinese medicine has a good clinical effect in the treatment of LDH [17, 18]. Among them, Duhuo Jisheng Decoction (DHJSD) is a TCM recorded in Bei Ji Qian Jin Yao Fang in the Tang Dynasty [19]. According to the theory of TCM, DHJSD is mainly used to treat joint pain syndrome, which has the effects of eliminating stagnation, removing blood stasis, nourishing the kidney and liver, and invigorating Qi and blood [20].

DHJSD is usually composed of 15 commonly used herbs: Duhuo (Radix Angelicae Pubescentis), Danggui (Radix Angelicae Sinensis), Sangjisheng (Herba Taxilli), Duzhong (Cortex Eucommiae), Fangfeng (Radix Saposhnikoviae), Xixin (Herba Asari), Chuanxiong (Rhizoma Chuanxiong), Baishao (Radix Paeoniae Alba), Dihuang (Radix Rehmanniae Glutinosae), Rougui (Cortex Cinnamomi), Renshen (Panax Ginseng), Fuling (Poria), Niuxi (Radix Achyranthis Bidentatae), Qinjiao (Radix Gentianae Macrophyllae), and Gancao (Radix Glycyrrhizae) [20]. Each herb has its own unique medicinal value. Combination application can be beneficial, avoid harm, and play a more significant therapeutic role [16]. Many studies have shown that, on the basis of DHJSD, increasing or reducing the types of herbs can increase the efficacy and safety of DHJSD in the treatment of LDH [3–16]. At this time, the prescription changed to modified DHJSD. However, the efficacy and safety of modified DHJSD in the treatment of LDH remains controversial. We conducted a meta-analysis of randomized controlled trials (RCTs) to assess the efficacy and safety of modified DHJSD in the treatment of LDH, providing a reference for clinical practice.

## 2. Methodology

We carried out this meta-analysis according to the Preferred Reporting Items for Systematic Reviews and Meta-Analyses (PRISMA) statement [21].

### 2.1. Search Strategy

In order to obtain all the literature related to our research, first of all, two researchers independently used the keywords combined with free words to search multiple databases according to Cochrane Collaboration guidelines, such as PubMed (1966 to June 1, 2020), Embase (1990 to June 1, 2020), the Cochrane Library (1990 to June 1, 2020), China National Knowledge Infrastructure (CNKI) databases (1990 to June 1, 2020), Wanfang Database (1990 to June 1, 2020), and Chinese Scientific Journal Database (VIP) (1990 to June 1, 2020). Next, potentially related literature was searched from a list of references in all included studies. We searched for the following terms “Duhuo Jisheng Decoction or DHJSD,” “lumbar disc herniation,” “herniated disc or disk,” and “disc or disk, herniated” with the Boolean operators “AND or OR” using Medical Subject Headings (MeSH) terms and corresponding keywords. The corresponding Chinese translation of the search strategy was used for the Chinese database search. Then, two researchers independently screened the above-retrieved literature by reading the titles and abstracts. Finally, the selected literature was further filtered by reading the full text. After discussion, all disagreeable literature was resolved.

### 2.2. Study Selection

All trials included in our study meet the following criteria: (1) All patients included in these RCTs were diagnosed with LDH based on symptoms, signs, and imaging features. (2) All included studies were original RCTs. (3) In all included studies, the experimental group received modified DHJSD, while the control group received Western medicine. (4) Studies were published in Chinese or English. (5) The full text of the included literature can be obtained, and the measurement data of total effective rate, cure rate, and visual analogue scale (VAS) scores can be extracted.

The following studies were excluded from the meta-analysis: (1) Studies were not RCTs. (2) Patients had lumbar spinal stenosis, lateral recess stenosis, spinal tumors, tuberculosis, and ankylosing spondylitis. (3) The experimental group did not simply take modified DHJSD, combined with other drugs or treatments. (4) The patients took other drugs during treatment. (5) Studies full text and related data could not be obtained.

### 2.3. Data Extraction

Data were extracted independently by two researchers. After discussion, disagreements in the data extraction process were resolved by the two researchers, and then another researcher used the spreadsheet to collect the data. We extracted the following data: first author, publication year, country, study type, number of participants (modified DHJSD: control), age, gender, intervention (modified DHJSD: control), and treatment duration. Outcome measurements include total effective rate, cure rate, and VAS scores. Total effective rate refers to the ratio of the effective number of patients to the total number of patients. Cure rate refers to the ratio of the number of cured patients to the total number of people. VAS scores are used for pain assessment. A 10 cm horizontal line was drawn on the paper. One end of the horizontal line is 0, indicating no pain; the other end is 10, indicating severe pain; the middle part indicates different degrees of pain. Total effective rate and cure rate are the primary outcome measurements. VAS scores are the secondary outcome measurements. For dichotomous data, such as total effective rate and cure rate, we extracted the number of events and total number of patients in modified DHJSD group and the control group. For continuous data, such as VAS scores, we extracted mean, standard deviation (SD), and total number of patients in each group.

### 2.4. Quality Assessment

The risk of bias in each included RCT was assessed according to the Cochrane Handbook for Systematic Reviews [22]. The evaluation of bias can be divided into 7 sections: random sequence generation, allocation concealment, blinding of participant and personnel, blinding of outcome assessment, incomplete outcome data, selective reporting, and other bias. Each section can have a high, low, and unclear risk of bias depending on the actual content of the included study [22].

### 2.5. Statistical Analysis

Different studies compared modified DHJSD and Western medicine groups in the treatment of LDH according to the total effective rate, cure rate, and VAS scores, as well as adverse events. We pooled and calculated data for the same outcome measurement in all studies and placed them on the same form. The total effective rate and cure rate can be used for subgroup analysis according to the difference in Western medicine. We analyzed dichotomous data, such as the total effective rate and cure rate, using risk ratio (RR) and their 95% confidence interval (CI). We analyzed continuous data, such as VAS scores, using weighted mean differences (WMD) and their 95% CI. The units of total effective rate, cure rate, and VAS scores are numbers, so the units of measurements are standardized to make the meta-analysis feasible. Statistical heterogeneity was calculated using a chi-square test and *I*^2^ test. It is considered that the *I*^2^ values of 25%, 50%, and 75% indicate low, moderate, and high heterogeneity, respectively [23]. When *I*^2^ > 50% and *P* < 0.1, we performed a random-effect model; otherwise, a fixed-effect model was performed. Funnel plot was usually used to assess publication bias and was usually only performed in at least 10 studies [17]. The number of studies included will have an effect on the effectiveness of the funnel plot to test publication bias. If too few studies are included, the funnel plot's testing power will decrease accordingly. The meta-analysis was performed using RevMan 5.3 for Windows (Cochrane Collaboration, Oxford, UK). If the result of the meta-analysis was a probability of *P* < 0.05, it was considered statistically significant.

## 3. Results

### 3.1. Study Selection

**Figure 1:**
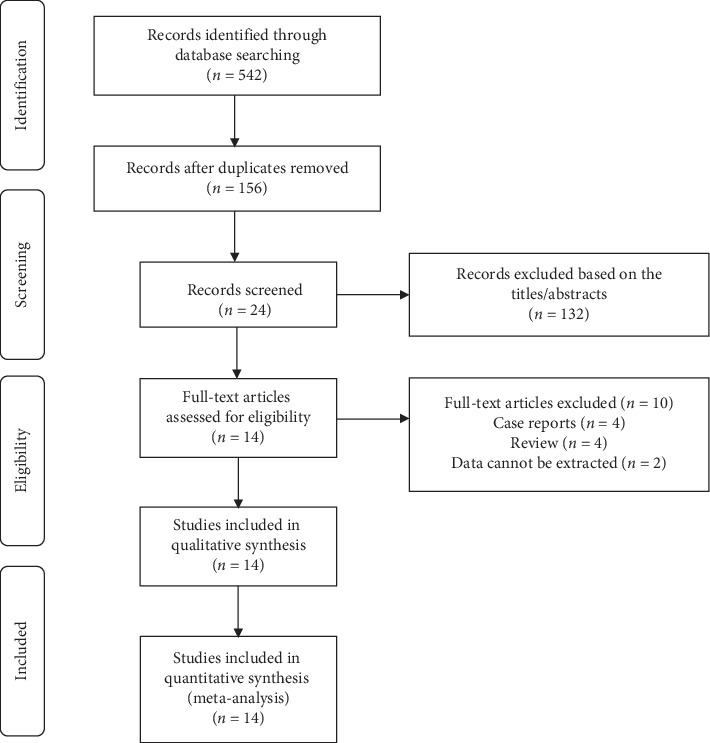
Flow diagram of the study selection process for the meta-analysis.

Firstly, we searched multiple databases using keywords and free words and finally confirmed 542 records. Then, a total of 24 records were screened out by reading titles and abstracts to remove duplicate records and irrelevant records. According to the inclusion criteria, records of non-RCTs, letters, or reviews and records whose data could not be extracted were excluded. Finally, by reading the full text, a total of 14 RCTs were selected [3–16]. No additional studies were identified from the reference lists.  Figure 1 shows the search strategy and the process of the study selection.

### 3.2. Study Characteristics

This meta-analysis included a total of 14 RCTs published between 2009 and 2019 [3–16]. Characteristics of all the studies included in the meta-analysis are shown in Table 1. All studies focused on the efficacy and safety of modified DHJSD in the treatment of LDH compared with Western medicine. In these studies, the number of patients in the modified DHJSD group (784 patients) was higher than that in the Western medicine group (776 patients) [3–16]. In eleven studies, the number of male patients (797 patients) was greater than the number of female patients (431 patients) [3–5, 7, 9–14, 16]. In fourteen studies, all research teams were from China [3–16]. In a total of 3 studies [3, 7, 15], patients were treated for 20 days, and in another 3 studies [5, 8, 16], they were treated for 21 days. Table 1 shows that all patients included in the 14 RCTs were randomly assigned to receive modified DHJSD or Western medicine [3–16]. In all studies, the types and frequency of Chinese herbal medicines in the modified DHJSD were not completely consistent, and the Western medicine groups were also different. A total of 3 studies provided outcomes measures with VAS scores [13, 15, 16] and adverse events [3, 5, 6], and 14 studies [3–16] provided the total effective rate and cure rate.

All included studies were based on criteria of diagnosis and therapeutic effect of diseases and syndromes in traditional Chinese medicine to evaluate the efficacy of drugs, especially Chinese medicine, in the treatment of LDH [3–16] as follows: cure: the waist and leg pain disappear completely, and the straight leg is raised to 70° or more; significant effect: the waist and leg pain are obviously reduced, the waist activity is close to normal, and the straight leg is raised above 60°, less than 70°; effectiveness: the symptoms of the waist and leg pain disappear partially and the functional activities improve; invalidity: symptoms and signs are the same as before.

### 3.3. Risk of Bias

**Figure 2:**
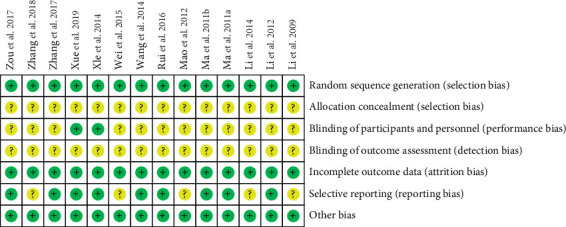
Risk of bias summary: +, low risk of bias; −, high risk of bias;?, bias unclear.

Random sequence generation was found in 8 studies [3, 4, 7, 8, 10, 13, 15, 16]. Allocation concealment and blinding of outcome assessment were not found in all studies. Blinding of participants and personnel was found in 2 studies [8, 16]. A total of 9 studies did not report selectively [4–6, 8, 9, 12–14, 16]. As shown in Figure 2, none of the fourteen studies found incomplete results data and other bias [3–16].

### 3.4. Results of the Meta-Analysis

After carefully reading and analyzing the included articles, we summarized the outcome measurements used to assess the efficacy and safety of modified DHJSD and Western medicine, including total effective rate, cure rate, and VAS scores.

#### 3.4.1. Total Effective Rate

**Figure 3:**
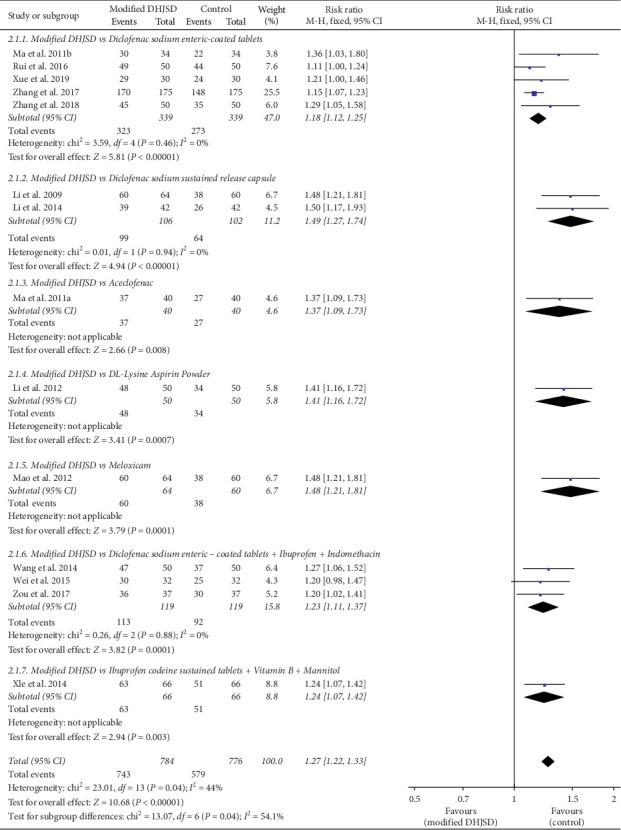
Forest plot showing the effect of modified DHJSD versus different Western medicines on the total effective rate in the treatment of LDH (DHJSD: Duhuo Jisheng Decoction; LDH: lumbar disc herniation; CI: confidence interval; M-H: Mantel–Haenszel).

Fourteen RCTs used the total effective rate as the primary outcome measurement [3–16]. As shown in Figure 3, the forest plot shows a subgroup analysis of the total effective rate of modified DHJSD and different Western medicines in the treatment of LDH. Regarding modified DHJSD versus diclofenac sodium enteric-coated tablets, in 5 RCTs [5, 12, 14–16], the subgroup analysis showed a significant difference (RR = 1.18, 95% CI: 1.12 to 1.25, *P* < 0.00001, *I*^2^ = 0%). For modified DHJSD versus diclofenac sodium sustained-release capsule, in two RCTs [3, 10], the subgroup analysis suggested a significant difference (RR = 1.49, 95% CI: 1.27 to 1.74, *P* < 0.00001, *I*^2^ = 0%). Concerning modified DHJSD versus diclofenac sodium enteric-coated tablets plus ibuprofen and indomethacin, the subgroup included three RCTs, which showed a significant difference (RR = 1.23, 95% CI: 1.11 to 1.37, *P*=0.0001, *I*^2^ = 0%) [9, 11, 13]. In addition, individual RCTs compared modified DHSJD with aceclofenac, DL-Lysine Aspirin Powder, meloxicam, and ibuprofen codeine sustained tablets plus vitamin B_1_ and mannitol, all of which also demonstrated a higher total effective rate in the modified DHJSD group [4, 6–8]. *I*^2^ = 0% means that the three studies are highly homogenous, and the pooled analysis results of the data are meaningful.

#### 3.4.2. Cure Rate

**Figure 4:**
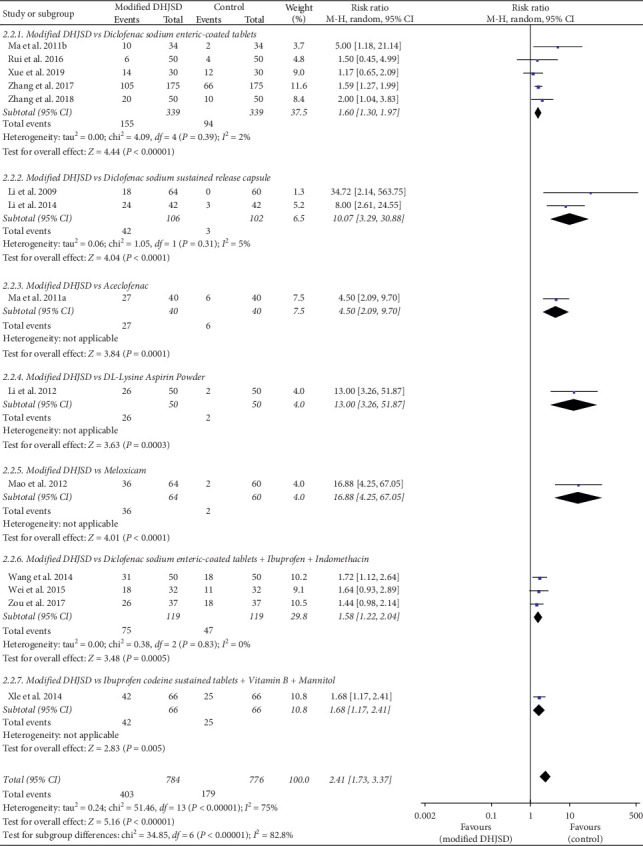
Forest plot showing the effect of modified DHJSD versus different Western medicines on the cure rate in the treatment of LDH.

Fourteen RCTs used the cure rate as the primary outcome measurement [3–16]. As shown in Figure 4, the forest plot shows a subgroup analysis of the cure rate of modified DHJSD and different Western medicines in the treatment of LDH. As to modified DHJSD versus diclofenac sodium enteric-coated tablets, in 5 RCTs [5, 12, 14–16], the subgroup analysis showed a significant difference (RR = 1.60, 95% CI: 1.30 to 1.97, *P* < 0.00001, *I*^2^ = 2%). As regards modified DHJSD versus diclofenac sodium sustained-release capsule, in two RCTs [3, 10], the subgroup analysis suggested a significant difference (RR = 10.07, 95% CI: 3.29 to 30.88, *P* < 0.0001, *I*^2^ = 5%). As for modified DHJSD versus diclofenac sodium enteric-coated tablets plus ibuprofen and indomethacin, the subgroup included three RCTs, which showed a significant difference (RR = 1.58, 95% CI: 1.22 to 2.04, *P*=0.0005, *I*^2^ = 0%) [9, 11, 13]. In addition, individual RCTs compared modified DHSJD with aceclofenac, DL-Lysine Aspirin Powder, meloxicam, and ibuprofen codeine sustained tablets plus vitamin B_1_ and mannitol, all of which also demonstrated a higher cure rate in the modified DHJSD group [4, 6–8].

#### 3.4.3. VAS Scores

**Figure 5:**
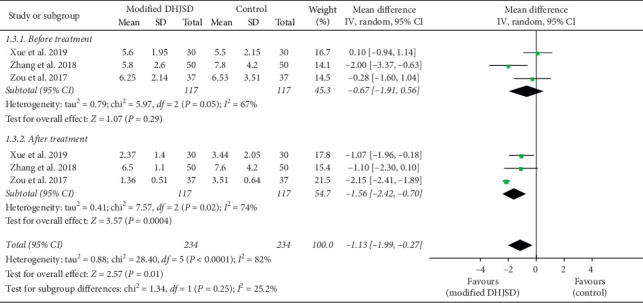
Forest plot showing the effect of modified DHJSD versus Western medicine on VAS scores in the treatment of LDH (VAS, visual analogue scale; IV, inverse variance).

Three RCTs used VAS scores as the secondary outcome measurements [13, 15, 16]. As shown in Figure 5, the forest plot shows a comparison of the VAS scores between the modified DHJSD group and the Western medicine group before and after treatment. A total of 3 studies (234 patients) provided data on VAS scores for modified DHJSD and Western medicine before treatment [13, 15, 16]. Based on the results of the pooled analysis, there were no statistically significant differences between the two groups at the VAS scores (MD = -0.67, 95% CI: −1.91 to 0.56, *P*=0.29, *I*^2^ = 67%). In addition, a total of 3 studies (234 patients) provided data on VAS scores for modified DHJSD and Western medicine after treatment [13, 15, 16]. Based on the results of the pooled analysis, there was a statistically significant difference between the two groups at the VAS scores (MD = −1.56, 95% CI: −2.42 to −0.70, *P*=0.0004, *I*^2^ = 74%). When *I*^2^ > 50%, this means that the included studies are highly heterogeneous. The heterogeneity of the above results is high and may be related to the inclusion of too few studies, requiring more high-quality RCTs. The above results indicated a larger effect in the modified DHJSD group in terms of VAS scores.

#### 3.4.4. Adverse Events

Three RCTs reported adverse events [3, 5, 6]. One RCT stated that there were no adverse events in the modified DHJSD group, and four patients in the control group reported stomach discomfort and dizziness after oral administration of diclofenac sodium sustained-release capsules [3]. One RCT stated that there were no adverse events in the modified DHJSD group, but two patients reported stomach discomfort and dizziness after oral administration of diclofenac sodium enteric-coated tablets [5]. Finally, one RCT stated that there were no adverse events in the modified DHJSD group, but in the control group, 5 patients had nausea, vomiting, and upper abdominal pain after oral administration of DL-Lysine Aspirin Powder [6].

### 3.5. Publication Bias

**Figure 6:**
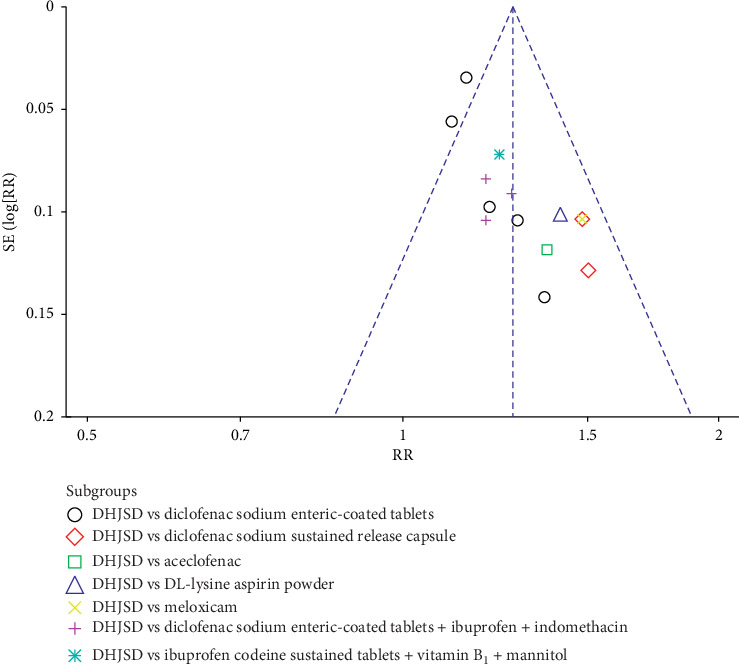
Funnel plot to detect publication bias for studies comparing the effect of modified DHJSD versus different Western medicines on the total effective rate in the treatment of LDH.

As shown in Figure 6, we used the funnel plot to detect publication bias for studies comparing the effect of modified DHJSD versus different Western medicines on the total effective rate in the treatment of LDH. No significant funnel asymmetry that could indicate publication bias was observed.

### 3.6. Sensitivity Analysis

If necessary, a sensitivity analysis was conducted to identify the origins of the significant heterogeneity. Due to the high heterogeneity of the VAS score before and after treatment, we performed a sensitivity analysis to assess the reliability of the results. However, there were only three studies that met the inclusion criteria, and the reliability of the results might be affected by the limited number of studies included.

## 4. Discussion

LDH is a common clinical disease, which is easy to recur and difficult to cure thoroughly and effectively [1]. LDH is a symptom of lumbar pain, nerve numbness, and weakness caused by degeneration of intervertebral disc, protrusion of nucleus pulposus, rupture of fibrous ring, and compression of nerve root and cauda equina nerve [14, 16]. Usually, most patients with LDH can be treated conservatively [1]. Drug treatment is a widely used conservative treatment, including Western medicine and Chinese medicine. In recent years, Chinese scholars have conducted a lot of research and found that modified DHJSD can effectively improve inflammation, edema of nerve roots and surrounding tissues, and pain [3–16].

According to TCM theory, fifteen main Chinese herbs in DHJSD play important roles, respectively [3–16]. Among them, Duhuo is the basic herbal medicine, which plays the most important role. Duhuo is good at dispelling wind, cold, and dampness evil between “Xia Jiao,” tendons, and bones [3]. “Xia Jiao” is the internal organs below the umbilical cord, including the kidney, large intestine, small intestine, and bladder [24]. Xixin relieves pain by dispelling Yin meridian and wind-cold, searching for bones, muscles, and rheumatism [4]. Fangfeng dispels wind evil to wind dampness [5]. Qinjiao eliminates rheumatism and relaxes tendons [6]. Sangjisheng, Duzhong, and Niuxi dispel rheumatism and supplement liver and kidney [7]. Danggui, Chuanxiong, Dihuang, and Baishao can nourish blood and promote blood circulation [8]. Renshen and Fuling supplement Qi and strengthen spleen [9]. Rougui warms and dredges blood vessels [10]. Gancao plays a role in reconciling these TCMs [11]. Modified DHJSD will increase or decrease the type of Chinese herbal medicine under the following conditions. If the heat is too heavy, the same amount of Chishao (Radix Paeoniae Rubra) is added as Baishao, and the Shudihuang (Rehmannia glutinosa) is replaced with Shengdihuang (Dry Radix Rehmannia) [12]. If the cold evil is heavy, dried ginger or Paofuzi (Radix Aconiti Praeparata) can be increased [13]. If the dampness evil is heavy, Cangzhu (Atractylodes Lancea) or Fangji (Stephania tetrandra) can be increased [14]. Patients with blood stasis can increase Honghua (Carthamus tinctorius) and Taoren (Peach Kernel) [15]. Patients with obvious numbness can increase Huangqi (Astragalus membranaceus) [16]. For patients with aggravated low back pain, centipedes, scorpions, and Jixueteng (Caulis spatholobi) can be added [16]. The Chinese herbal medicine in modified DHJSD will be given with the corresponding dose and frequency of use according to the condition of each patient. The combination of these herbal medicines can dispel pathogenic factors and strengthen health, making blood and Qi sufficient, rheumatism eliminated, liver and kidney strong, and pain alleviated [3–16].

There is evidence that DHJSD has been widely used to treat a variety of diseases including osteoarthritis (OA), LDH, and rheumatoid arthritis [3–16, 20, 25]. Liu et al. [26] demonstrate that DHJSD inhibits tunicamycin-induced chondrocyte endoplasmic reticulum stress by downregulating miR-34a, suggesting that DHJSD may be a potential therapeutic agent for OA. In one study, Liu et al. [27] indicate that DHJSD inhibits sodium nitroprusside-induced chondrocyte apoptosis via a mitochondria-dependent apoptotic pathway, which may partly explain its therapeutic effect on OA. Wu et al. [28] indicate that DHJSD promotes chondrocyte proliferation by promoting G1/S checkpoint transition in the cell cycle, upregulation of cyclin D1, CDK4, CDK6, and Rb expression, and downregulation of p16 expression, which may partly explain the clinical efficacy in treating OA. However, the specific mechanism of DHJSD treatment of LDH is still controversial, and more relevant research is still needed.

A total of 14 studies (1560 patients) provided data on modified DHJSD and Western medicine [3–16]. Each study compared the efficacy and safety of modified DHJSD with one or more Western medicines for the treatment of LDH. The total effective rate was the primary outcome measurement. We conducted a subgroup analysis of the difference in Western medicine. A total of 5 studies (678 patients) [5, 12, 14–16] provided data on modified DHJSD versus diclofenac sodium enteric-coated tablets, 2 studies (208 patients) [3, 10] provided data on modified DHJSD versus diclofenac sodium sustained-release capsule, and 3 studies (238 patients) [9, 11, 13] provided data on modified DHJSD versus diclofenac sodium enteric-coated tablets plus ibuprofen and indomethacin. The remaining subgroups are single studies [4, 6–8]. Based on the results of the pooled analysis, there was a statistically significant difference between the two groups. The *P* value indicated that modified DHJSD had a higher total effective rate on LDH than Western medicine. The cure rate was outcome measurement. The subgroup of cure rates, as well as the number of people, was the same as the total effective rate. There were statistically significant differences between the two groups based on the results of the pooled analysis. The *P* value indicated that modified DHJSD had a higher cure rate on LDH than Western medicine. In 3 studies, VAS score was also the outcome measurement [13, 15, 16]. We performed a subgroup analysis of treatment time. A total of 3 studies (234 patients) provided data on VAS scores for modified DHJSD and Western medicine before and after treatment [13, 15, 16]. According to the pooled analysis of results before and after treatment, modified DHJSD may reduce the VAS scores more effectively than Western medicine. Moreover, in the studies we included, no adverse events were found in the modified DHJSD group [3, 5, 6]. However, the number and quality of related studies included are limited, and more high-quality RCTs are needed to refine this conclusion.

### 4.1. Limitations

This meta-analysis still has some limitations. First, most studies lacked details of random sequence generation, allocation concealment, blinding of participants and personnel, and blinding of outcome assessment. Second, many studies lacked details of long-term follow-up and adverse events. Third, the dose, frequency, and composition of modified DHJSD were not exactly the same, and the dose of the same Western medicine was not completely consistent. Fourth, the evaluation criteria for the effect after LDH treatment were not completely uniform. Fifth, for the pooled analysis of VAS scores, there was high heterogeneity. Sixth, all trials were from China and might have an impact on the conclusions. Finally, we used the total effective rate as the primary outcome measurement, which was an indicator that was not commonly used internationally. Therefore, more high-quality RCTs need to be conducted in the future. By studying the mechanism of DHJSD in the treatment of LDH, it can be more effective and safer.

## 5. Conclusion

The oral treatment of TCM is simple, easy to implement, economical, and easy to accept and has a remarkable curative effect. It is worth promoting in clinical practice. The results of the above analysis indicated that modified DHJSD had a more favorable effect on the treatment of LDH than Western medicine, and there were no obvious adverse events. However, due to the generally low or very low quality of the included trials, further rigorous design requires large RCTs to confirm the current conclusions.

## Figures and Tables

**Table 1:** Characteristics of all the trials included in the meta-analysis.

Authors	Country	Study type	Sample size D : C	Mean age (years) D : C	Gender M : F	Intervention	Treatment duration (days)
Li and He [3]	China	RCTs	124 (64/60)	38.5/37.6	114/10	3 times, 100 mL, every day^†^ DSSRC, 1 time, 100 mg, every day^‡^	20^†^ 20^‡^
Li [6]	China	RCTs	100 (50/50)	52.2/53.1	46/54	2 times, every day^†^ DL-LAP, 4 times, 900 mg, every day^‡^	NP^†^ NP^‡^
Li [10]	China	RCTs	84 (42/42)	49.5/49.5	49/35	3 times, 100 mL, every day^†^ DSSRC, 1 time, 100 mg, every day^‡^	NP^†^ NP^‡^
Ma [4]	China	RCTs	80 (40/40)	48.5/48	45/35	3 times, 250 mL, every day^†^ aceclofenac, 2 times, 100 mg, every day^‡^	30^†^ 20^‡^
Ma et al. [5]	China	RCTs	68 (34/34)	48.3/48.3	41/27	2 times, every day^†^ DSECT, 3 times, 25 mg, every day^‡^	21^†^ 21^‡^
Mao [7]	China	RCTs	124 (64/60)	39.5/38.6	98/26	3 times, 100 mL, every day^†^ meloxicam, 1 time, every day^‡^	20^†^ 20^‡^
Rui and Zhao [12]	China	RCTs	100 (50/50)	38.8/39.4	80/20	NP^†^ DSECT, 1 time, 100 mg, every day^‡^	NP^†^ 28^‡^
Wang and Yang [9]	China	RCTs	100 (50/50)	NP	65/35	2 times, every day^†^ 3 times, DSECT, 25 mg; ibuprofen, 200 mg; indomethacin, 25 mg, every day^‡^	15^†^ 15^‡^
Wei and Yang [11]	China	RCTs	64 (32/32)	41.5/43.2	38/26	2 times, every day^†^ NP^‡^	28^†^ NP^‡^
Xie and Du [8]	China	RCTs	132 (66/66)	NP	NP	3 times, every day^†^ 2 times, ICST, 213 mg; mannitol, 250 ml; vitamin B_1_, 10 mg, every day^‡^	21^†^ 21^‡^
Xue [16]	China	RCTs	60 (30/30)	40.5/40.2	37/23	2 times, every day^†^ DSECT, 3 times, 25 mg, every day^‡^	21^†^ 21^‡^
Zhang et al. [14]	China	RCTs	350 (175/175)	42.8/41.7	189/161	2 times, every day^†^ DSECT, 1 time, 100 mg, every day^‡^	14^†^ 14^‡^
Zhang and Zhong [15]	China	RCTs	100 (50/50)	NP	49/51	3 times, 250 mL, every day^†^ DSECT, 2 times, 100 mg, every day^‡^	20^†^ 20^‡^
Zou [13]	China	RCTs	74 (37/37)	55.2/55.4	41/33	1 time, every day^†^ NP^‡^	30^†^ 30^‡^

DHJSD: Duhuo Jisheng Decoction; RCTs: randomized controlled trials; DSSRC: diclofenac sodium sustained-release capsule; DSECT: diclofenac sodium enteric-coated tablets; DL-LAP: DL-Lysine Aspirin Powder; ICST: ibuprofen codeine sustained tablets; NP: not provided. ^†^D: DHJSD group, ^‡^C: control group.

## Data Availability

The data supporting this meta-analysis were obtained from previously reported studies and datasets, which have been cited.

## Supplementary Materials

Supplementary Materials The retrieval strategy for each database is included in the supplementary materials.
