# Supplementary material for: Efficacy and Safety of Modified Duhuo Jisheng Decoction in the Treatment of Lumbar Disc Herniation: A Systematic Review and Meta-Analysis
Source: Evid Based Complement Alternat Med. 2020 Jul 4;2020:2381462. doi: 10.1155/2020/2381462 (PMC7355353; doi:10.1155/2020/2381462)
Supplement: Supplementary Materials — The retrieval strategy for each database is included in the supplementary materials. [file 2381462.f1.docx]

***Search strategy***

***Pubmed***

*(lumbar disc herniation) AND (Duhuo Jisheng Decoction) AND ("1966/01/01"[PDat]: "2020/06/01"[PDat])*

*Result: 2*

***Embase***

*#1 'duhuo jisheng decoction' AND 'lumbar disk hernia' AND [1990-2020]/py*

*Result: 1*

***Cochrane library***

*3 Trials matching Duhuo Jisheng Decoction in All Text AND lumbar disc herniation in All Text - with Cochrane Library publication date Between Jan 1990 and Jun 2020 (Word variations have been searched)*

*Result: 3*

***China National Knowledge Infrastructure databases (CNKI)***

*#1. 独活寄生汤*

*#2. 腰椎间盘突出*

*#3. 联合*

*#4. 配合*

*#5. 结合*

*#1 and #2 not #3* *not #4* *not #5*

*Result: 144*

**WanFang Database**

*#1. 独活寄生汤*

*#2. 腰椎间盘突出*

*#3. 联合*

*#4. 配合*

*#5. 结合*

*#1 and #2 not #3 not #4 not #5*

*Result: 218*

**Chinese Scientific Journal Database (VIP)**

*#1. 独活寄生汤*

*#2. 腰椎间盘突出*

*#3. 联合*

*#4. 配合*

*#5. 结合*

*#1 and #2 not #3* *not #4* *not #5*

*Result: 175*
